# Analytical Solution for Thermal Buckling of Functionally Graded Graphene Origami-Enabled Auxetic Metamaterial Cylindrical Shells

**DOI:** 10.3390/nano16150917

**Published:** 2026-07-26

**Authors:** Zuoquan Zhu, Nan Zhao, Yuyan Zhou, Jianfeng Lu

**Affiliations:** 1 School of Mathematics and Statistics, Zhengzhou Normal University, Zhengzhou 450044, China; 2 School of Mechanical Engineering, Suzhou University of Technology, Changshu 215500, China

**Keywords:** graphene origami metamaterial, cylindrical shell, geometric nonlinearity, temperature-dependent, critical buckling temperature

## Abstract

Composite cylindrical shells suffer from thermal buckling in harsh thermal environments, impairing overall structural safety. This study aims to improve the thermal stability of such shells by investigating the thermal buckling behavior of graphene origami metamaterial-reinforced composite cylindrical shells. Four common thickness-wise distribution patterns (UD, FG-X, FG-O, and FG-A) are adopted, and temperature-dependent material properties are taken into account. Based on classical thin-shell theory with geometric nonlinearity, thermal buckling governing equations are derived. Analytical solutions of critical buckling temperature rises are obtained via an iterative procedure for both temperature-dependent and temperature-independent material models. Parametric studies are conducted to explore key influencing factors including reinforcement distribution, filler content, folding degree, tangential edge constraints, and shell geometric parameters. The results reveal that critical buckling temperature is strongly dependent on graphene origami distribution and structural features. Increasing filler content enhances thermal buckling resistance, while folding degree also dominates structural stability. Additionally, tangential constraints and geometric dimensions exert obvious effects. Significant discrepancies exist between two material models, verifying that temperature-dependent material properties are essential for precise thermal buckling analysis of the proposed composite shells.

## 1. Introduction

As the core load-bearing structural units, beams, plates, and shells derive their structural performance primarily from buckling behavior, which, in turn, directly determines the safety margin, load-bearing efficiency, and service life of the entire structure [1,2,3,4]. Cylindrical shells represent a prevalent class of structures, extensively employed in aerospace engineering, marine engineering, nuclear engineering, pressure vessel systems, and a broad range of other engineering applications. These structures typically operate under severe thermal and mechanical loading conditions. Consequently, the accurate prediction of buckling stability and load-carrying capacity for such shell (and plate) structures is critical to realizing reliable structural designs.

Functionally graded materials (FGMs) have emerged as a novel class of composite structures characterized by continuously varying material properties. When incorporated into cylindrical shells, such gradient distributions provide significant engineering advantages by enhancing structural performance under complex loading conditions. Theoretically, classical shell theory combined with Donnell’s simplified assumptions has established the fundamental framework for analyzing cylindrical shell mechanics [5]. With the advancement of research on FGM shell structures, higher-order shear deformation theory (HSDT) has gradually become a widely adopted approach for investigating their mechanical behavior [6,7,8]. Shen et al. [9,10] studied the post-buckling behavior of FGM cylindrical shells under axial compression using second-order perturbation theory and demonstrated that the gradient material distributions can significantly enhance buckling resistance. She et al. [11,12] investigated the thermal post-buckling behavior of graphene platelet-reinforced metal foam cylindrical shells with geometric imperfections. Sofiyev et al. [13] systematically revealed the influence of functionally graded orthotropy on the buckling performance of FGM cylindrical shells by solving the nonlinear von Kármán equations. These theoretical studies show that an optimized ceramic–metal power-law gradient can increase the critical buckling load of FGM cylindrical shells by approximately 15–30% compared with homogeneous materials while maintaining excellent thermomechanical performance. Further investigations have considered multi-field coupling and advanced numerical techniques. Mirzavand et al. [14,15] conducted thermal buckling analyses of laminated cylindrical shells with surface-bonded piezoelectric actuators under combined thermal–electrical loading. Liew et al. [16] used the meshless method to trace the post-buckling equilibrium path and identified snap-through instability phenomenon in FGM cylindrical shells. Safarpour et al. [17] addressed the buckling problems under complex boundary conditions based on the differential quadrature method. Lu et al. [18,19] introduced the nonlocal elasticity theory into the buckling analysis of FGM cylindrical shells, providing new insights into nanoscale structural behavior. From an experimental perspective, early studies on the buckling of FGM cylindrical shells were limited due to constraints in traditional fabrication techniques [20,21]. With the rapid development of additive manufacturing technologies, Xiong et al. [22] successfully fabricated FGM cylindrical shells based on the Ti6Al4V/SiC system with high precision. Xin et al. further verified the theoretical predictions results through the digital image correlation technique [23]. Arbelo [24] developed a non-destructive vibration-driven technique to estimate the actual buckling load of cylindrical shells under compressive loading. Beyond validating theoretical models, these experimental investigations also reveal the significant influence of practical factors-–such as manufacturing defects and microstructural characteristics-–on the buckling behavior of FGM cylindrical shells, emphasizing their critical importance for structural safety assessment.

On the other hand, functional materials exhibiting negative Poisson’s ratio (NPR) effects, particularly those associated with graphene origami (GOri)-reinforced structures, have attracted increasing attention due to their unique mechanical characteristics and tunable deformation mechanisms. At the microscale, Zhao et al. [25,26] revealed the auxetic mechanism of GOri/Cu nanocomposites through molecular dynamics simulations, demonstrating that the negative Poisson’s ratio of the nanocomposite with 3.35 wt% Miura-patterned GOri can induce the value –0.2796 at room temperature. Furthermore, combined with the genetic programming (GP) algorithm, a micromechanical model capable of accurately predicting the properties of functionally graded graphene origami-enabled auxetic metamaterials (FG-GOEAMs), such as the elastic modulus and coefficient of thermal expansion, was established, providing a reliable foundation for subsequent macrostructural analysis. Building upon these micromechanical insights, extensive studies have been conducted to explore the structural behavior and tunability of FG-GOEAMs, primarily focusing on beam and plate configurations. For instance, the thermal buckling behavior of FG-GOEAM beams resting on Winkler–Pasternak elastic foundations was investigated using refined beam theory and Hamilton’s principle, revealing that GOri distribution patterns, content, folding degree, and foundation parameters play significant roles in regulating the critical buckling load, with the Pasternak shear layer contributing more effectively to stability enhancement [27]. In addition, the dynamic characteristics of multilayer FG-GOEAM plates were analyzed based on refined sinusoidal plate theory, where the influences of temperature, GOri distribution, and folding degree on natural frequencies were systematically quantified, demonstrating superior dynamic performance compared with conventional materials [28,29,30]. From the perspective of active control, Ebrahimi and Ahari [31] incorporated magnetostrictive layers into FG-GOEAM beams and, employing HSDT in conjunction with nonlocal elasticity theory, highlighted the dual role of GOri reinforcement in enhancing natural frequencies and improving vibration suppression efficiency under thermal gradients. Morever, Lv et al. [32] investigated the mechanical and thermal post-buckling behavior of FG-GOEAM plates under biaxial compression and uniform thermal loading using first-order shear deformation theory and a two-step perturbation method, confirming that GOri distribution patterns and elastic foundation parameters can effectively tailor both the critical buckling load and thermal stability. Recently, Arshid et al. [33,34] developed a computational framework is to investigate the thermal buckling behavior of doubly curved shells reinforced with FG-GOEAM. Overall, these studies have systematically advanced the understanding of FG-GOEAMs, spanning from microscale deformation mechanisms and property prediction to macroscale structural performance and control strategies, thereby establishing a solid foundation for their application in extreme environments. However, despite these significant developments, investigations on the thermal buckling behavior of graphene origami-reinforced composite cylindrical shells remain very limited, particularly when accounting for temperature-dependent material properties and complex geometric configurations. On the other hand, it is a fact that no analytical investigation on the nonlinear stability of FG-GOEAM cylindrical shells, taking tangential edge restraint into consideration, has been reported in the literature.

This paper aims to investigate the thermal buckling behavior of graphene origami-reinforced composite cylindrical shells. Based on the von Kármán–Donnell geometrically nonlinear thin-shell theory, the governing equations for the buckling analysis of such cylindrical shells are established. In the thermal stability analysis, both tangential boundary constraints and temperature-dependent material properties are taken into account. Considering that the structure is subjected to a uniformly heated environment, a uniform temperature rise is assumed for the thermal loading condition. An admissible approximate solution is first introduced, and the Galerkin method, combined with an iterative procedure, is employed to derive analytical solutions for the buckling characteristics of the cylindrical shells. Finally, a comprehensive parametric study is conducted to systematically examine the effects of graphene origami characteristics, geometric parameters, and tangential boundary constraints on the nonlinear stability of the cylindrical shells.

## 2. Graphene Origami Metamaterial Composite Cylindrical Shell

**Figure 1:**
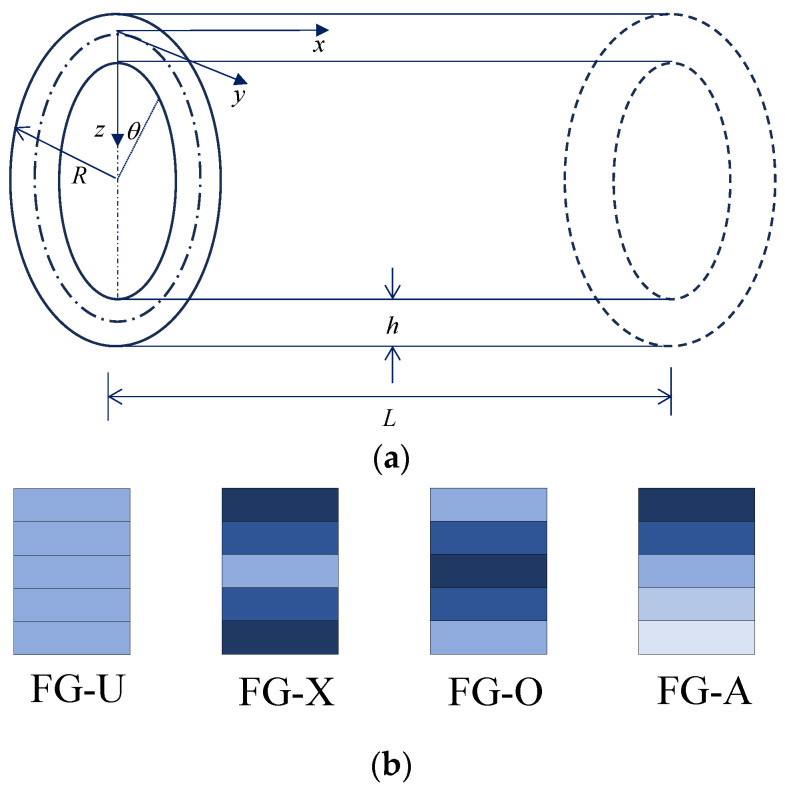
(**a**) Geometric structure of graphene origami-reinforced composite cylindrical shell; (**b**) various distribution types of graphene origami for an auxetic metamaterial shell.

Consider a functionally graded GOri/Cu composite cylindrical shell with length L, thickness h, and curvature radius R, as shown in Figure 1a. The shell is composed of a Cu matrix and a graphene origami structure and is defined by a coordinate system$\;\left(x,\theta ,z\right)$. The origin of this coordinate system is located on the mid-surface of the shell, where the *x*-axis and *θ*-axis extend along the axial and circumferential directions, respectively, and the *z*-axis is perpendicular to the mid-surface and points to the inner side of the shell (with a value range of $-\frac{h}{2}\leq z\leq \frac{h}{2}$). It is assumed that the shell structure is isotropic and linearly elastic, and its material properties distribute along a gradient in the thickness direction, as shown in Figure 1b. Here, the FG-U composite shell shows the same NPR behavior in every layer. In contrast, for the FG-X shell, a larger NPR is observed in the outer layers, whereas the central layer exhibits a positive Poisson’s ratio. For the FG-A shell, the Poisson’s ratio gradually decreases from the top layer, where it is positive, to the bottom layer, where it becomes negative. The folding degree $H_{GR}$ of graphene origami is determined by the H atom coverage (i.e., hydrogenation degree, ranging from 0% to 100%). The folding-degree parameter characterizes the geometric deformation of graphene origami. Increasing the folding degree compresses origami-unit dimensions and enhances the auxetic effect. Accordingly, the overall Poisson’s ratio and equivalent elastic modulus of graphene origami-reinforced composites change considerably, which further governs the critical buckling temperature of the cylindrical shell. Meanwhile, four types of graphene origami distribution forms are considered, corresponding to the volume fractions $V_{GR}^{\left(k\right)}$ of different graphene distribution patterns across a single layer, indexed from k=1 to $N_{L}$ (where the index represents the total number of layers in the origami), which are defined as follows:

$$U\text{-}W_{\mathrm{GR}}\text{:}\;\;\;\;V_{GR}^{\left(k\right)}=V_{GR}$$

$$X\text{-}W_{\mathrm{GR}}\text{:}\;\;\;\;V_{GR}^{\left(k\right)}=2V_{GR}\frac{\left|2k-N_{L}-1\right|}{N_{L}}$$

(1) $$O\text{-}W_{\mathrm{GR}}\text{:}\;\;\;\;V_{GR}^{\left(k\right)}=2V_{GR}\left(1-\frac{\left|2k-N_{L}-1\right|}{N_{L}}\right)$$

$$A\text{-}W_{\mathrm{GR}}\text{:}\;\;\;\;V_{GR}^{\left(k\right)}=\frac{V_{GR}\left(2k-1\right)}{N_{L}}$$

Among them, $V_{GR}$ is the total volume fraction of GOri, which can be obtained using the formula of mass fraction $W_{GR}$:

(2) $$V_{GR}=\frac{W_{GR}}{W_{GR}+\left(\frac{\rho_{GR}}{\rho_{Cu}}\right)\left(1-W_{GR}\right)}$$

In Equation (2), $W_{GR}$ is the total weight fraction of GOri, $\rho_{GR}$ is the density of graphene, and $\rho_{Cu}$ is the density of Cu. It is worth noting that the volume fractions of both GOri and the Cu matrix are linked to one another as:

(3) $$V_{Cu}=1-V_{GR}$$

The equivalent elastic modulus ($E_{e}$), Poisson’s ratio ($v_{e}$), and thermal expansion coefficient ($\alpha_{e}$) of the composite material in terms of mass fraction and density are determined by the micromechanical model assisted by genetic programming (GP) proposed by Zhao et al. [25,26]:

$$E_{e}=\frac{1+\xi \lambda V_{GR}}{1-\lambda V_{GR}}E_{Cu}\times f_{E}\left(H_{GR},V_{GR},T\right)$$

$$v_{e}=\left(v_{GR}V_{GR}+v_{Cu}V_{Cu}\right)\times f_{v}\left(H_{GR},V_{GR},T\right)$$

(4) $$\alpha_{e}=\left(\alpha_{GR}V_{GR}+\alpha_{Cu}V_{Cu}\right)\times f_{\alpha }\left(V_{GR},T\right)$$

The material coefficient (λ) and size coefficient (ξ) are as follows:

(5) $$\lambda =\frac{\left(\frac{E_{GR}}{E_{Cu}}\right)-1}{\left(\frac{E_{GR}}{E_{Cu}}\right)+\xi },\xi =2\left(\frac{l_{GR}}{t_{GR}}\right)$$

Among them, $l_{GR}$ and $t_{GR}$ denote the length and thickness of the GOri, respectively. The transformation functions $f_{E,v,\rho }\left(H_{GR},V_{GR},T\right)$ were derived by the genetic programming proposed by Zhao et al. [25,26]:

$$f_{E}\left(H_{GR},V_{GR},T\right)=1.11-1.22V_{GR}-0.134\left(\frac{T}{T_{0}}\right)+0.559V_{GR}\left(\frac{T}{T_{0}}\right)-5.5H_{GR}V_{GR}+38H_{GR}V_{GR}^{2}-20.6H_{GR}^{2}V_{GR}^{2}$$

$$f_{v}\left(H_{GR},V_{GR},T\right)=1.01-1.43V_{GR}+0.165\left(\frac{T}{T_{0}}\right)-16.8H_{GR}V_{GR}-1.1H_{GR}V_{GR}\left(\frac{T}{T_{0}}\right)+16H_{GR}^{2}V_{GR}^{2}$$

(6) $$f_{\alpha }\left(V_{GR},T\right)=1.01-2.01V_{GR}^{2}-0.0131\left(\frac{T}{T_{0}}\right)$$

**Figure 2:**
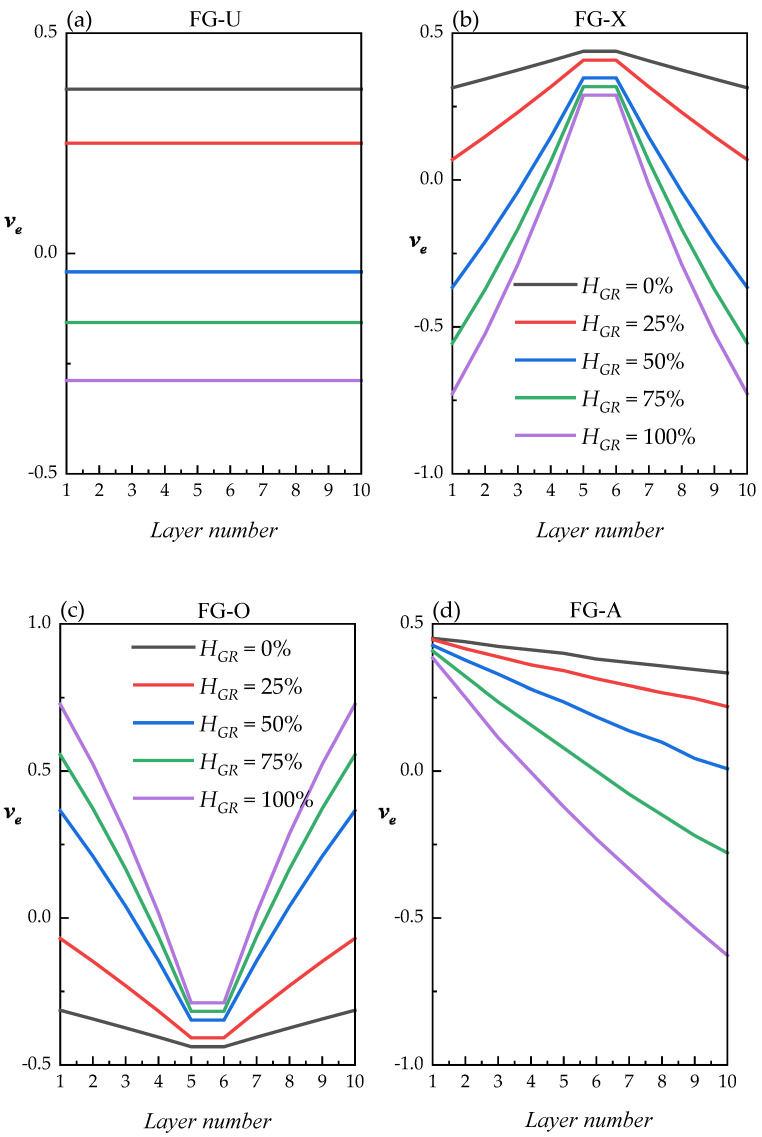
Effective Poisson’s ratio values of FG-GOEAM cylindrical shells along the thickness for (**a**) FG-U, (**b**) FG-X, (**c**) FG-O, and (**d**) FG-A under GOri patterns and various GOri folding degrees (W_GR_ = 2.5 wt%).

Set the temperature $T_{0}$ to 300 K, and denote the current ambient temperature as T. Figure 2 shows the equivalent Poisson’s ratio distribution of the cylindrical shell structure under different folding degrees when the graphene mass fraction is 2.5 wt% for four distribution forms. It is evident that as the number of layers increases from 1 to 10, more and more layers in the FG shells exhibit negative Poisson’s ratio behavior with increasing GOri content and degree of folding. Therefore, the composite beam can be regarded as a metamaterial beam when an NPR layer is present in the FG shell.

## 3. Fundamental Equations

In this study, the fundamental equations for the buckling of geometrically perfect graphene origami metamaterial composite cylindrical shells are established. Following the classical thin-shell theory (*R*/*h* ≫ 1), transverse shear deformation is neglected, and normals to the mid-surface are assumed to remain straight and normal during deformation. Therefore, the in-plane linear strain components $\varepsilon_{x}$,$\;\varepsilon_{y}$, and the mid-surface shear strain $\gamma_{xy}$ are expressed as [5]:

(7) $$\left(\begin{array}{c} \varepsilon_{x}\\ \varepsilon_{y}\\ \gamma_{xy} \end{array}\right)=\left(\begin{array}{c} \varepsilon_{x}^{0}\\ \varepsilon_{y}^{0}\\ \gamma_{xy}^{0} \end{array}\right)+z\left(\begin{array}{c} \chi_{x}\\ \chi_{y}\\ 2\chi_{xy} \end{array}\right)$$

where

(8) $$\left(\begin{array}{c} \varepsilon_{x}^{0}\\ \varepsilon_{y}^{0}\\ \gamma_{xy}^{0} \end{array}\right)=\left(\begin{array}{c} u_{,x}+\frac{w_{,x}^{2}}{2}\\ v_{,y}-\frac{w}{R}+\frac{w_{,y}^{2}}{2}\\ u_{,y}+v_{,x}+w_{,x}w_{,y} \end{array}\right),\;\left(\begin{array}{c} \chi_{x}\\ \chi_{y}\\ \chi_{xy} \end{array}\right)=\left(\begin{array}{c} -w_{,xx}\\ -w_{,yy}\\ -w_{,xy} \end{array}\right)$$

Among them, u, v, and w are the displacement components in the x, y, and z  directions, respectively, while $\chi_{x}$ and $\chi_{y}$ are the curvatures of the mid-surface normal relative to the x-axis and y-axis, respectively; $\chi_{xy}$ is the torsion, and y=Rθ. Here, the subscript comma denotes the partial derivative with respect to the subsequent variable, for example, $u_{,x}=\partial u/\partial x$.

The stress components in the graphene origami composite cylindrical shell are expressed as follows:

$$\sigma_{x}=\frac{E_{e}}{1-{\nu_{e}}^{2}}\left[\varepsilon_{x}+\nu_{e}\varepsilon_{y}-\left(1+\nu_{e}\right)\alpha_{e}\Delta T\right]$$

$$\sigma_{y}=\frac{E_{e}}{1-{\nu_{e}}^{2}}\left[\varepsilon_{y}+\nu_{e}\varepsilon_{x}-\left(1+\nu_{e}\right)\alpha_{e}\Delta T\right]$$

(9) $$\sigma_{xy}=\frac{E_{e}}{2\left(1+\nu_{e}\right)}\gamma_{xy}$$

Among them, $\Delta T=T-T_{0}$ is the value at which the shell is free of thermal stress under uniform temperature rise starting from the initial temperature $T_{0}$.

The membrane forces $N_{x}$, $N_{y}$, and $N_{xy}$, bending moments $M_{x}$ and $M_{y}$ per unit length, and torque $M_{xy}$ are defined as follows:

$$\left(N_{x},N_{y},N_{xy}\right)=\int_{-\frac{h}{2}}^{\frac{h}{2}}\left(\sigma_{x},\sigma_{y},\sigma_{xy}\right)dz$$

(10) $$\left(M_{x},M_{y},M_{xy}\right)=\int_{-\frac{h}{2}}^{\frac{h}{2}}\left(\sigma_{x},\sigma_{y},\sigma_{xy}\right)zdz$$

Meanwhile, substituting Equations (7) and (9) into Equation (10) yields

$$\left(N_{x},M_{x}\right)=\frac{1}{1-{\nu_{e}}^{2}}\left[\begin{array}{c} \left(A_{11},B_{11}\right)\left(\varepsilon_{x}^{0}+\nu_{e}\varepsilon_{y}^{0}\right)\\ +\left(B_{11},D_{11}\right)\left(\chi_{x}+\nu_{e}\chi_{y}\right) \end{array}\right]-\frac{1}{1-\nu_{e}}\left(N_{T},M_{T}\right)$$

$$\left(N_{y},M_{y}\right)=\frac{1}{1-{\nu_{e}}^{2}}\left[\begin{array}{c} \left(A_{11},B_{11}\right)\left(\varepsilon_{y}^{0}+\nu_{e}\varepsilon_{x}^{0}\right)\\ +\left(B_{11},D_{11}\right)\left(\chi_{y}+\nu_{e}\chi_{x}\right) \end{array}\right]-\frac{1}{1-\nu_{e}}\left(N_{T},M_{T}\right)$$

(11) $$\left(N_{xy},M_{xy}\right)=\frac{1}{2\left(1+\nu_{e}\right)}\left[\left(A_{11},B_{11}\right)\gamma_{xy}^{0}+2\left(B_{11},D_{11}\right)\chi_{xy}\right]$$

where

$$\left(A_{11},B_{11},D_{11}\right)=\int_{-\frac{h}{2}}^{\frac{h}{2}}E_{e}\left(1,z,z^{2}\right)dz$$

(12) $$\left(N_{T},M_{T}\right)=\int_{-\frac{h}{2}}^{\frac{h}{2}}E_{e}\alpha_{e}\Delta T\left(1,z\right)dz$$

The equilibrium equations of the cylindrical shell under thermal loading are as follows [5]:

(13a) $$M_{x,xx}+2M_{xy,xy}+M_{y,yy}+\frac{N_{y}}{R}+N_{x}w_{,xx}+2N_{xy}w_{,xy}+N_{y}w_{,yy}=0$$

(13b) $$N_{x,x}+N_{xy,y}=0$$

(13c) $$N_{xy,x}+N_{y,y}=0$$

A stress function $f\left(x,y\right)$ is introduced, which satisfies $N_{x}=f_{,yy}$, $N_{y}=f_{,xx}$, and $N_{xy}=-f_{,xy}$. Substituting Equation (11) into Equation (13a), and using Equations (8) and (11), the nonlinear equilibrium equation of the cylindrical shell is expressed in a compact form as

(14) $$D\nabla^{4}w-f_{,yy}w_{,xx}+2f_{,xy}w_{,xy}-f_{,xx}w_{,yy}-\frac{f_{,xx}}{R}=0$$

Among them, D is the bending stiffness, and $\nabla^{2}$ is the Laplacian operator, which is defined as follows:

(15) $$D=\frac{A_{11}D_{11}-B_{11}^{2}}{A_{11}\left(1-{\nu_{e}}^{2}\right)},\;\nabla^{2}=\frac{\partial^{2}}{\partial x^{2}}+\frac{\partial^{2}}{\partial y^{2}}$$

According to Equation (6), $\varepsilon_{x}^{0}$, $\varepsilon_{y}^{0}$, and $\gamma_{xy}^{0}$ can be expressed in terms of the partial derivatives of the stress function $f\left(x,y\right)$. Therefore, the strain compatibility equation can be written as follows:

(16) $$\nabla^{4}f-A_{11}\left(w_{,xy}^{2}-\frac{w_{,xx}}{R}-w_{,xx}w_{,yy}\right)=0$$

Among them, $\nabla^{4}=\nabla^{2}\nabla^{2}$ is the biharmonic operator.

In the present work, the two edges of the GOri-Cu composite cylindrical shell are assumed to be simply supported and elastically constrained in the tangential direction. The relevant boundary conditions are expressed as follows:

(17) $$w=M_{x}=N_{xy}=0,N_{x}=N_{x0}\;\mathrm{at}\;x=0,L$$

Among them, $N_{x0}$ is the virtual pressure resultant force generated by the tangential constraint at the boundary edge, which is related to the average end shortening displacement as follows:

(18) $$N_{x0}=-\frac{c}{2\pi RL}\int_{0}^{2\pi R}\int_{0}^{L}\frac{\partial u}{\partial x}dxdy$$

Among them, *c* denotes the average tangential stiffness of the edge constraint. As is well known, the values of c→∞ and 0<c<∞ represent the fully immovable and partially movable boundary conditions, respectively.

For cylindrical shells, the condition of a closed circumference must satisfy the following universal condition, which is expressed as follows:

(19) $$\frac{1}{2\pi RL}\int_{0}^{2\pi R}\int_{0}^{L}\frac{\partial v}{\partial y}dxdy=0$$

Solving Equations (8) and (11) for u and v, we obtain

(20a) $$\frac{\partial u}{\partial x}=\frac{1}{A_{11}}\left(f_{,yy}-\nu_{e}f_{,xx}\right)+\frac{B_{11}}{A_{11}}w_{,xx}-\frac{1}{2}w_{,x}^{2}+\frac{N_{T}}{A_{11}}$$

(20b) $$\frac{\partial v}{\partial y}=\frac{1}{A_{11}}\left(f_{,xx}-\nu_{e}f_{,yy}\right)+\frac{B_{11}}{A_{11}}w_{,yy}+\frac{w}{R}-\frac{1}{2}w_{,y}^{2}+\frac{N_{T}}{A_{11}}$$

## 4. Solution Method

To solve the buckling governing equations, as stated in the work by Brush and Almroth [5], unlike plate structures, the pre-buckling deformation of cylindrical shells is quite significant and should be considered in the stability analysis. Based on this practical basis, the approximate solution for the buckling of simply supported cylindrical shells is assumed to take the following form:

(21a) $$w\left(x,y\right)=W\sin\beta x\sin\delta y$$

(21b) $$f\left(x,y\right)=A_{1}\cos2\beta x+A_{2}\cos2\delta y+A_{3}\sin\beta x\sin\delta y+\frac{1}{2}N_{x0}y^{2}$$

Among them, W is the amplitude of the buckling deflection; $\beta =\frac{m\pi }{L}$, *m* is the number of axial half-waves, $\delta_{n}=\frac{n}{R}$, and *n* is the number of circumferential waves (*m*, *n* = 1, 2,…). In addition, in Equation (21a,b), $A_{i}$ (i=1−3) are undetermined coefficients. By substituting the solutions Equation (21a,b) into the strain compatibility Equation (16), the coefficients$\;A_{i}$ (i=1−3) can be obtained as follows:

(22) $$A_{1}=\frac{A_{11}\delta^{2}}{32\beta^{2}}W^{2},A_{2}=\frac{A_{11}\beta^{2}}{32\delta^{2}}W^{2},A_{3}=\frac{A_{11}\beta^{2}W}{R{\left(\beta^{2}+\delta^{2}\right)}^{2}}$$

Subsequently, substituting solution Equation (21) into equilibrium Equation (14), applying the Galerkin method over the entire domain of the cylindrical shell (0≤x≤L, 0≤y≤2πR), and considering the condition for a non-trivial solution (i.e., W≠0), we obtain the following system of equation:

(23) $$\left[D{\left(\beta^{2}+\delta^{2}\right)}^{2}+\frac{A_{11}\beta^{4}}{R^{2}{\left(\beta^{4}+\delta^{4}\right)}^{2}}+\beta^{2}N_{x0}\right]W+\frac{A_{11}\left(\beta^{4}+\delta^{4}\right)}{16}W^{3}=0\;$$

Substituting Equation (20a) into Equation (18), $N_{x0}$ can be expressed as:

(24) $$N_{x0}=\frac{A_{11}\eta \beta^{2}}{8}W^{2}-\eta N_{T}$$

where

(25) $$\eta =\frac{c}{A_{11}+c},\;G=\frac{1}{h}\int_{-\frac{h}{2}}^{\frac{h}{2}}E_{e}\alpha_{e}dz,\;N_{T}=Gh\Delta T$$

Now, substituting Equation (24) into Equation (23), the following expression for the pre-buckling deflection is obtained:

(26) $$\Delta T=\left[\frac{\bar{D}{\left(m^{2}\pi^{2}+n^{2}L_{R}^{2}\right)}^{2}}{\eta Gm^{2}\pi^{2}R_{h}^{2}L_{R}^{2}}+\frac{{\bar{A}}_{11}m^{2}\pi^{2}L_{R}^{2}}{\eta G{\left(m^{2}\pi^{2}+n^{2}L_{R}^{2}\right)}^{2}}\right]+\left[\frac{{\bar{A}}_{11}\left(m^{4}\pi^{4}+n^{4}{\bar{L}}_{R}^{4}\right)}{16\eta Gm^{2}\pi^{2}R_{h}^{2}L_{R}^{2}}+\frac{{\bar{A}}_{11}m^{2}\pi^{2}}{8GR_{h}^{2}L_{R}^{2}}\right]{\bar{W}}^{2}\;$$

Among them, $\bar{W}=\frac{W}{h}$ $\bar{D}=\frac{D}{h^{3}},{\bar{A}}_{11}=\frac{A_{11}}{h}$, the radius-to-thickness ratio *R*_*h*_ = *R/h*, and the length-to-radius ratio *L*_*R*_ = *L/R*. The complete expression of Equations (23)–(26) can be found in Appendix A. Finally, from Equation (26), by letting $\bar{W}\to 0$, the critical buckling temperature rise can be obtained as follows:

(27) $$\Delta T_{b}=\frac{{\bar{A}}_{11}m^{2}\pi^{2}L_{R}^{2}}{\eta G{\left(m^{2}\pi^{2}+n^{2}L_{R}^{2}\right)}^{2}}+\frac{\bar{D}{\left(m^{2}\pi^{2}+n^{2}L_{R}^{2}\right)}^{2}}{\eta Gm^{2}\pi^{2}R_{h}^{2}L_{R}^{2}}$$

The critical buckling temperature rise ${\Delta T_{cr}}^{\prime \prime }$ is the minimum value $\Delta T_{b}$ relative to the buckling mode (*m*, *n*). Furthermore, it is noteworthy that if η = 0 (i.e., c=0), then $N_{x0}=0$, and the buckling temperature change $\Delta T_{b}$ tends to infinity. This indicates that, as expected, graphene origami-reinforced cylindrical shells with freely movable edges do not buckle under uniform temperature rise, similar to other porous FGM cylindrical shell structures [7,35,36]. To predict the critical buckling temperature for temperature-dependent material properties, the iterative process involves the following steps: (1) First, using the temperature-independent material properties (T-ID properties) calculated at the reference temperature $T_{0}$, solve for the buckling temperature $\Delta T_{cr}\;$ with minimization of $\Delta T_{b}$ with respect to ${\left(m^{2}\pi^{2}+n^{2}L_{R}^{2}\right)}^{2}$ via Equation (27). (2) Update the right-hand side of Equation (27) using the property values at temperature $T=T_{0}+\Delta T_{b}$ to obtain a new buckling temperature. (3) Step (2) is repeated until the critical buckling temperature satisfies the prescribed convergence criterion. Specifically, convergence is achieved when the relative difference between two successive solutions is smaller than a specified tolerance: $\frac{\left|{\Delta T}_{cr}^{\left(k\right)}-{\Delta T}_{cr}^{\left(k-1\right)}\right|}{\left|{\Delta T}_{cr}^{\left(k\right)}\right|}$ ≤ *ψ*, where *k* denotes the iteration number, and *ψ* is the prescribed tolerance. In this paper, for the sake of brevity, temperature-dependent material properties and temperature-independent material properties are denoted as T-D properties and T-ID properties, respectively. Closed-form analytical solutions of the above-mentioned equations are obtained using Wolfram Mathematica (Version 12.1) with the iteration tolerance 10^−4^. The value denotes the convergence tolerance used to terminate the iterative numerical calculations in our solving program.

## 5. Results and Discussion

### 5.1. Validation

To ensure the accuracy and validity of the established analytical expression for the critical buckling temperature, a comparative study will be conducted in this section.

**Figure 3:**
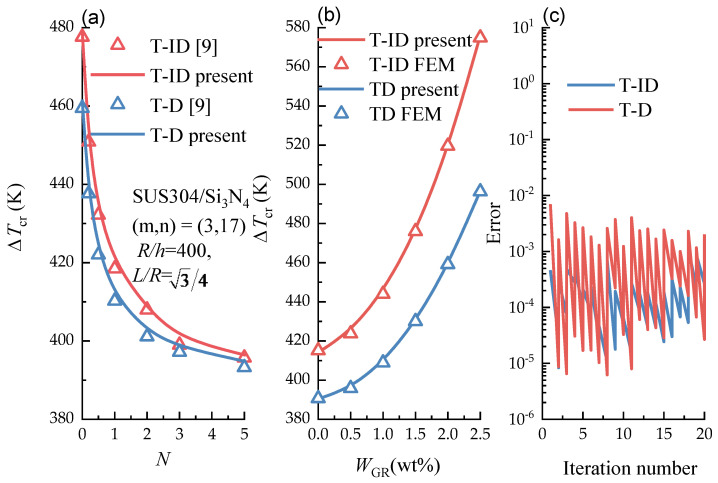
(**a**) Comparison of the buckling temperature of an FGM cylindrical shell; (**b**) comparison of the thermal buckling results of FG-U cylindrical shells with FEM; (**c**) the iteration error with the present method.

As part of the validation work for the proposed method, this section analyzes the buckling behavior of a thin functionally graded material (FGM) cylindrical shell with simply supported and fixed edges under uniform temperature rise; Shen also investigated this problem using classical shell theory and the singular perturbation method in the literature [9]. Figure 3a compares the calculation results of this paper with those of Reference [9], where the research object is an ideal cylindrical shell composed of silicon nitride and stainless steel (SUS304/Si_3_N_4_): the inner surface of the cylindrical shell is rich in ceramic phase, while the outer surface is rich in metal phase. Among them, the temperature-independent properties (T-ID properties) are calculated at room temperature, *T*_0_ =300 K. It can be seen from the data in the figure that the results of this comparative study are in good agreement. Moreover, we compare the results with the finite element method (FEM) data in Figure 3b. The FEM results were obtained via the commercial FEM software Abaqus 6.14 with S4R shell element. In the following, copper (Cu) is used as the metal matrix of the metamaterial, and its material property parameters at 300 K are as follows: Young’s modulus $E_{Cu\;}=65.79\;\mathrm{Gpa}$, Poisson’s ratio $\nu_{Cu}=0.387$, thermal expansion coefficient $\alpha_{Cu}=16.51\times 10^{-6}{\;K}^{-1}$, and mass density $\rho_{Cu}=\frac{8.80\;g}{{\mathrm{cm}}^{3}}$. Those of pristine graphene are $E_{GR}=929.57\;\mathrm{Gpa}$, $\nu_{GR}=0.220$, and $\alpha GR=-3.98\times 10^{-6}{\;K}^{-1}$, as well as its dimensions: length $l_{GR}=83.76\;\overset{\circ }{A}$ and thickness $t_{GR}=3.4\;\overset{\circ }{A}$, as reported in Refs. [25,26]. In this work, the GOri density $\rho_{GR\;}$ is set to 1.8 g/cm^3^ from Refs. [25,26] rather than the theoretical value 2.26 g/cm^3^. The difference comes from massive vacancies, oxygen-containing groups, and micro-voids inside reduced oxide graphene after powder-metallurgy preparation, which decreases its practical bulk density. The buckling response of the FG-U structure is obtained by using the nonlinear arc-length method. It is observed that the analytical predictions are in good agreement with the FEM results. Figure 3c presents the iteration-error history for the 2.5 wt% FG-U case. It can be observed that the desired converged results are obtained within fewer than 20 iteration steps. Convergence tests are conducted to determine suitable iteration parameter *k* and layer number *N_L_*. Table 1 lists the dimensionless critical-buckling and post-buckling loads of FG-GOEAM cylindrical shells for different *k* and *N_L_*. Results converge at *k* = 20 and *N_L_* = 50. Considering computational efficiency, *k* = 20 and *N_L_* = 10 are chosen for further simulations.

### 5.2. Parametric Analysis

Next, to further elaborate on the buckling instability of graphene origami-reinforced cylindrical shells, we will discuss in detail the effects of the distribution mode, content, and folding degree of graphene origami, as well as tangential boundary constraints and geometric dimensions on the critical buckling temperature.

**Figure 4:**
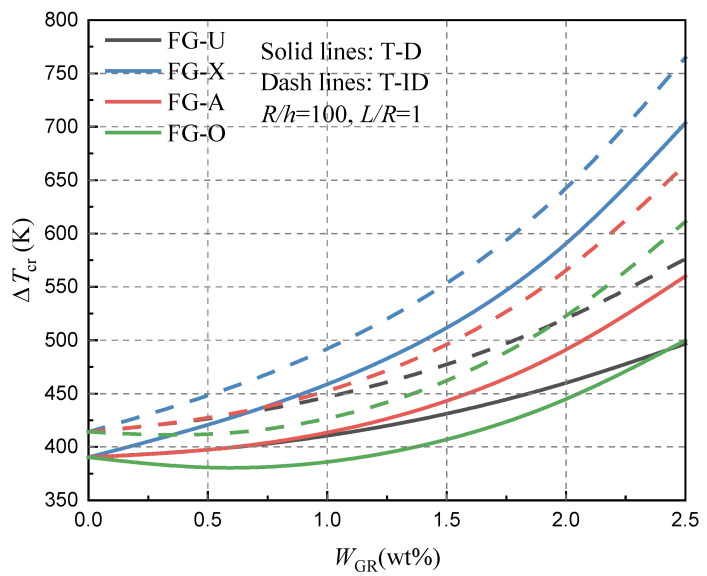
Variation of critical buckling load versus GOri content for four distribution patterns.

Figure 4 presents the buckling temperature-rise load characteristics of graphene origami-reinforced cylindrical shells. These cylindrical shells cover different distribution modes of graphene origami and take into account the correlation between graphene mass fraction and temperature. Two core laws can be clearly observed from the data in the Figure 4: First, the temperature dependence of material properties will significantly weaken the high-temperature resistance of graphene origami composite shells. Second, for all types of cylindrical shells, the graphene mass fraction has an obvious impact on the critical buckling temperature, and the buckling load increases significantly with the increase in GOri content, among which the cylindrical shell structure with X-type graphene mass fraction distribution (FG-X) shows the most prominent performance change. The reason lies in the uniform distribution of graphene in its outer layer: the uniform outer layer distribution structure endows the cylindrical shell with higher overall stiffness; in terms of the ranking of stiffness and load-bearing capacity, the cylindrical shells with A-type (FG-A), U-type (FG-U), and O-type (FG-O) graphene mass fraction distributions follow the FG-X structure in sequence.

**Figure 5:**
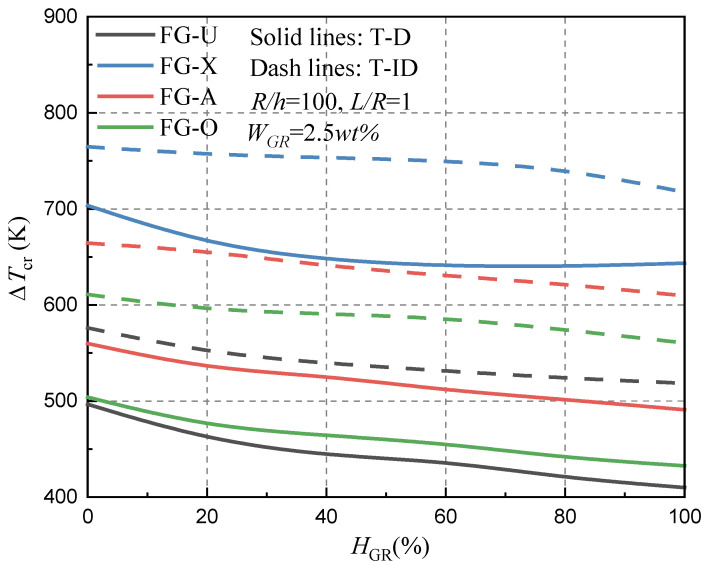
Variation of critical buckling temperature versus GOri folding degree in different distribution gradients.

Figure 5 illustrates the variation in the critical buckling temperature of functionally graded graphene origami-reinforced cylindrical shells with increasing graphene origami folding degree under different temperature gradients. As observed, the critical buckling temperature decreases as the folding degree increases. This trend can be attributed to the reduction in the effective elastic modulus and the emergence of a negative Poisson’s ratio effect. It is also evident that the FG-X cylindrical shell exhibits the highest critical buckling temperature, followed by the FG-A, FG-O, and FG-U configurations. Additionally, the buckling resistance decreases with increasing H_GR_. When temperature-dependent material properties are considered, the buckling load is further reduced due to the softening effect induced by elevated temperatures. Although the X and A patterns demonstrate relatively higher initial buckling loads, they are more sensitive to increases in H_GR_ compared to the FG-U and FG-O patterns. In contrast, the FG-U and FG-O configurations show greater stability, as their buckling performance is less affected by H_GR_ growth. Therefore, to achieve improved structural performance of such metamaterials, it is preferable to distribute a greater amount of graphene origami near the outer surfaces of the cylindrical shell.

**Figure 6:**
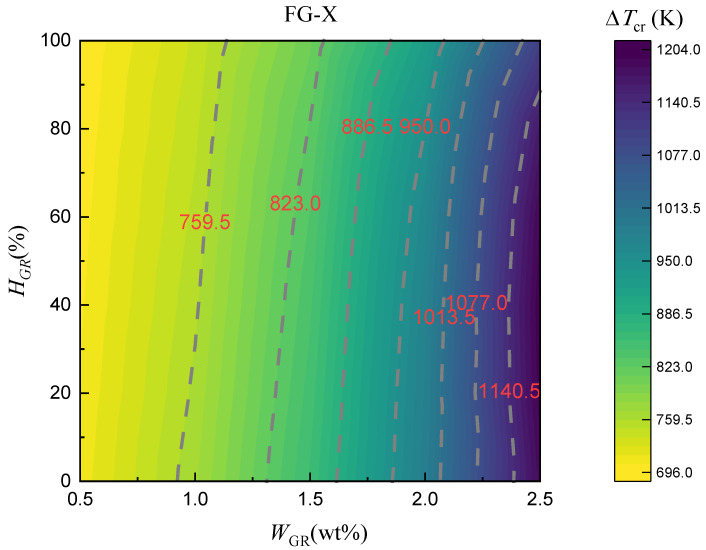
Design pattern based on buckling resistance of FG-X structure; the red numbers denote different critical buckling temperatures.

Considering the FG-X cylindrical shell structure with the strongest buckling resistance, Figure 6 presents an optimized design diagram based on the content and folding degree of graphene origami. In addition, it can be seen that there is a competitive mechanism between the content of graphene origami and the folding degree; that is, the higher the graphene content, the better the buckling resistance, and the greater the folding degree, the worse the buckling resistance, so the trade-off between them is needed to ensure state-of-the-art buckling performance. Furthermore, in conjunction with Figure 5, it can be observed that for a fixed folding degree, the buckling resistance increases with the parameter under consideration, indicating an enhancement in the effective bending stiffness of the cylindrical shell. During the design phase of metadevices, we can adjust the origami configuration and gradient distribution to reduce the critical buckling temperature and alleviate thermal-induced structural instability, which optimizes mechanical–thermal parameters.

**Figure 7:**
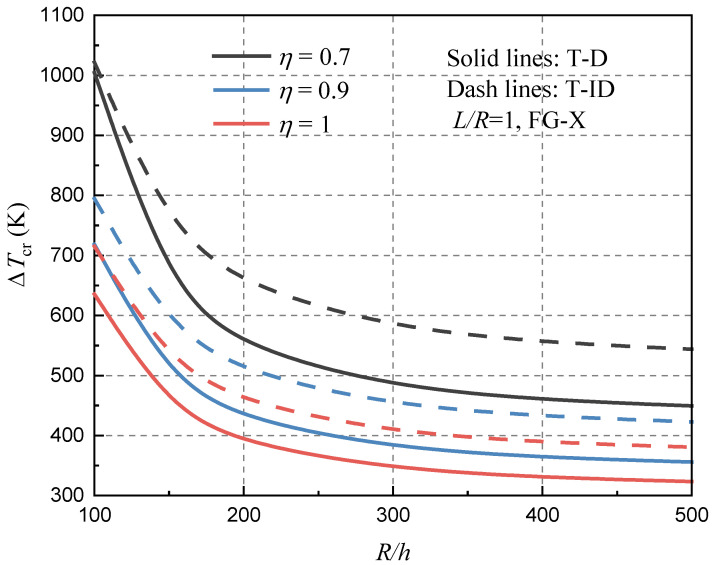
Effect of radius–thickness ratio R/h and tangential stiffness parameter on the critical buckling temperature.

Figure 7 describes the influence of geometric parameters and tangential stiffness coefficients on the critical buckling temperature. It reveals that the buckling temperature shows a nonlinear decreasing trend with an increase in the R/h ratio. The larger the diameter–thickness ratio, the lower the critical buckling load and the weaker the anti-instability capacity, and this correlation will be further intensified under conditions such as thin shells and temperature effects. Meanwhile, we also investigated the influence of tangential stiffness parameters η on the buckling behavior of ideal graphene origami-reinforced composite cylindrical shells under uniform temperature rise conditions. The results show that when the shell edge is in a partially movable state and the tangential stiffness parameter η is small, the thermal load-bearing capacity of the FGM cylindrical shell will be significantly improved. Apparently, η=0 and η=1 correspond to freely-movable and fully immovable edge conditions, respectively, and partial tangential constraints at *x* = 0 and *x* = *L* are defined for 0 < η < 1. Equation (27) demonstrates that $\Delta T_{b}$ approaches infinity when η→0. This finding indicates that cylindrical shells with completely movable edges can avoid thermal buckling under temperature rise, which is physically reasonable.

**Figure 8:**
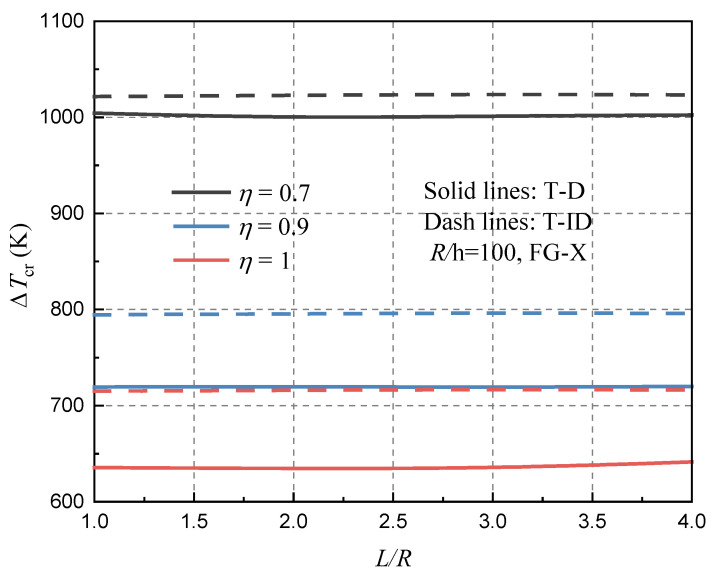
Effect of slenderness ratio L/R of shell geometry on the critical buckling temperature.

Figure 8 shows the influence of the length–diameter ratio L/R  on the critical buckling temperature of cylindrical shells. It can be seen from Figure 8 that the length–diameter ratio L/R  has a negligible effect on the buckling temperature. The reason is that when the edge of the cylindrical shell is elastically constrained (e.g., connected by spring supports) or partially movable (e.g., with a low tangential stiffness parameter), the stress generated by thermal expansion can be released through edge displacement, and the influence of the length–diameter ratio is significantly weakened. A similar trend has also been reported in the literature [37].

## 6. Conclusions

This paper presents an analytical approach for investigating the nonlinear stability of simply supported functionally graded graphene origami-reinforced cylindrical shells subjected to thermo-mechanical coupling loads. The governing formulations are derived from classical thin-shell theory by taking geometric nonlinearity, tangential boundary constraints, and temperature-dependent material properties into account, and the Galerkin method together with an iterative algorithm is adopted to solve buckling responses.

Even though the above theoretical framework follows conventional continuum-shell theory, some predicted characteristics are unique to graphene origami structures. The effects of the elastic modulus, boundary restraints, and filler fraction on thermal buckling are universal for shells reinforced by high-stiffness inclusions and have nothing to do with micro-structures. However, the origami-induced NPR effect belongs to our structural feature. When H GR > 25%, lateral expansion caused by the NPR effect further reduces the critical buckling temperature. Additionally, gradient-distributed origami cells achieve thickness-dependent Poisson ratio adjustment, which cannot be realized in shells with randomly dispersed graphene.

The results demonstrate that the graphene origami content, folding degree, temperature dependence of material properties, geometric parameters, and tangential stiffness coefficients significantly affect the buckling load and load-carrying capacity of graphene origami-reinforced composite cylindrical shells. The temperature dependence of material properties reduces the high-temperature resistance of graphene origami-reinforced composite shells, and this degradation becomes more pronounced for shells with an FG-O distribution pattern and partially constrained boundaries. Moreover, among the cylindrical shells subjected to thermal loads, the FG-X distribution pattern exhibits superior buckling resistance. These findings demonstrate that optimizing the distribution pattern of graphene origami is critical to enhancing the high-temperature resistance and structural stiffness of graphene origami-reinforced cylindrical shells. The obtained results can be extended to practical scenarios ranging from heat-resistant thin-walled components in aerospace systems to high-temperature-proof housings for semiconductor packaging. In future studies, benefiting from the von Kármán–Donnell shell theory and generalized gradient modeling concepts, our analytical framework goes beyond FG-GOEAM cylindrical shell problems. Once material distribution, geometry, and boundary conditions are properly adjusted, it works well for predicting nonlinear stability of origami-inspired metamaterial shells with different design parameters.

Nevertheless, the present study still has several inherent limitations worthy of discussion. Firstly, all calculations rely on classical thin-shell theory under *R*/*h* = 100. This theory is well suited for thin-wall cylinders, yet it is not applicable for moderately thick shells with reduced R/h. Secondly, we adopt the through-thickness uniform-temperature assumption, which fails when rapid heating occurs in actual service and produces through-thickness temperature gradients. Thirdly, the origami structure is treated as perfectly formed, while local folding defects and residual stresses are generated during preparation and weaken structural stability. Future work may extend the present framework by incorporating shear-deformable shell theories, examining the sensitivity of buckling responses to initial geometric imperfections, considering the effects of thermal gradients, and further investigating nonlinear post-buckling behavior.

## Figures and Tables

**Table 1:** Convergence studies on T-D buckling results with various numbers of iteration and layers for an FG-U cylindrical shell ($\frac{R}{h}=100,L/R=1,H_{GR}=100\%,{and\;W}_{GR}=2.5\;\text{wt\%}$).

*k*/*N_L_*	10	20	50
10	496.44906	495.86934	496.32715
15	496.53872	497.24106	496.91753
20	496.67846	496.90173	496.71428

## Data Availability

The original contributions presented in this study are included in the article. Further inquiries can be directed to the corresponding author.

## Appendix A

In the following, the complete expressions and derivation processes of Equations (23)–(26) are as follows:

Derivation of Equation (23).

The transverse displacement and Airy stress function are assumed as

$$w\left(x,y\right)=W\sin\beta x\sin\delta y$$

$$f\left(x,y\right)=A_{1}\cos2\beta x+A_{2}\cos2\delta y+A_{3}\sin\beta x\sin\delta y+\frac{1}{2}N_{x0}y^{2}$$

where

$$A_{1}=\frac{A_{11}\delta^{2}}{32\beta^{2}}W^{2},A_{2}=\frac{A_{11}\beta^{2}}{32\delta^{2}}W^{2},A_{3}=\frac{A_{11}\beta^{2}W}{R{\left(\beta^{2}+\delta^{2}\right)}^{2}}$$

Substituting the above expressions into the nonlinear equilibrium equation yields

$$D\nabla^{4}w-f_{,yy}w_{,xx}+2f_{,xy}w_{,xy}-f_{,xx}w_{,yy}-\frac{f_{,xx}}{R}=0$$

Using the Galerkin projection and the weighting function sinβxsinδy over the complete cylindrical domain 0 ≤ *x* ≤ *L*, 0 ≤ *y* ≤ 2*πR*, we can obtain the amplitude equation. Since all integrals containing odd powers of sinδy vanish over the closed circumference, the quadratic terms in W disappear. The resulting equation is

(A1) $$\left[D{\left(\beta^{2}+\delta^{2}\right)}^{2}+\frac{A_{11}\beta^{4}}{R^{2}{\left(\beta^{4}+\delta^{4}\right)}^{2}}+\beta^{2}N_{x0}\right]W+\frac{A_{11}\left(\beta^{4}+\delta^{4}\right)}{16}W^{3}=0\;\;\;\;\;\;\;$$

Derivation of Equation (24).

The elastic end-restraint condition is

$$N_{x0}=-\frac{c}{2\pi RL}\int_{0}^{2\pi R}\int_{0}^{L}\frac{\partial u}{\partial x}dxdy$$

where

$$\frac{\partial u}{\partial x}=\frac{1}{A_{11}}\left(f_{,yy}-\nu_{e}f_{,xx}\right)+\frac{B_{11}}{A_{11}}w_{,xx}-\frac{1}{2}w_{,x}^{2}+\frac{N_{T}}{A_{11}}$$

For integration over the complete circumference,

$$w_{x,x}=0,\;f_{,yy}-\nu_{e}f_{,xx}=N_{x0},\;\mathrm{and}\;w_{,x}^{2}=\frac{\beta^{2}W^{2}}{4}.$$

Therefore,

$$\frac{\partial u}{\partial x}=\frac{N_{x0}}{A_{11}}-\frac{\beta^{2}W^{2}}{8}+\frac{N_{T}}{A_{11}}$$

Substituting this result into the end-restraint relation and rearranging yields

(A2) $$N_{x0}=\frac{A_{11}\eta \beta^{2}}{8}W^{2}-\eta N_{T}.$$

Definition of the parameters in Equation (25).

The end-restraint parameter and the equivalent thermal stiffness are defined as

(A3) $$\eta =\frac{c}{A_{11}+c},\;G=\frac{1}{h}\int_{-\frac{h}{2}}^{\frac{h}{2}}E_{e}\alpha_{e}dz,\;N_{T}=\Delta T$$

Derivation of Equation (26).

For a nontrivial solution, Equation (23) can be divided by *W*. Substituting Equation (24) gives

$$\eta \beta^{2}N_{T}=D{\left(\beta^{2}+\delta^{2}\right)}^{2}+\frac{A_{11}\beta^{4}}{R^{2}{\left(\beta^{2}+\delta^{2}\right)}^{2}}+[\frac{A_{11}\eta \beta^{4}}{8}+\frac{A_{11}\left(\beta^{4}+\delta^{4}\right)}{16}{]W}^{2}\;\;\;.$$

We use $N_{T}$ = *Gh* ΔT and introduce the dimensionless parameters $\bar{W}=\frac{W}{h}$, $\bar{D}={\frac{D}{h}}^{3},{\bar{A}}_{11}=\frac{A_{11}}{h}$, $\beta =\frac{m\pi }{L}$, $\delta_{n}=\frac{n}{R\;},$ the radius-to-thickness ratio *R_h_* = *R/h*, and the length-to-radius ratio *L_R_* = *L/R*.

Thus, the temperature–deflection relation becomes

(A4) $$\Delta T=\left[\frac{\bar{D}{\left(m^{2}\pi^{2}+n^{2}L_{R}^{2}\right)}^{2}}{\eta Gm^{2}\pi^{2}R_{h}^{2}L_{R}^{2}}+\frac{{\bar{A}}_{11}m^{2}\pi^{2}L_{R}^{2}}{\eta G{\left(m^{2}\pi^{2}+n^{2}L_{R}^{2}\right)}^{2}}\right]+\left[\frac{{\bar{A}}_{11}\left(m^{4}\pi^{4}+n^{4}{\bar{L}}_{R}^{4}\right)}{16\eta Gm^{2}\pi^{2}R_{h}^{2}L_{R}^{2}}+\frac{{\bar{A}}_{11}m^{2}\pi^{2}}{8GR_{h}^{2}L_{R}^{2}}\right]{\bar{W}}^{2}\;$$

Under closed-circumference integration, Equation (26) contains no term linear in $\bar{W}$ because all contributions involving odd powers of the circumferential sine function vanish over 0 ≤ *y* ≤ 2*πR*.
